# Dog‐assisted therapy in the dental clinic: Part A—Hazards and assessment of potential risks to the health and safety of humans

**DOI:** 10.1002/cre2.240

**Published:** 2019-08-16

**Authors:** Anne M. Gussgard, J. Scott Weese, Arne Hensten, Asbjørn Jokstad

**Affiliations:** ^1^ Faculty of Health Sciences UiT The Arctic University of Norway Tromsø Norway; ^2^ Centre for Public Health and Zoonoses, Ontario Veterinary College University of Guelph Guelph ON Canada

**Keywords:** anxiety, dental staff, disease vectors, dogs

## Abstract

**Background:**

Dog‐assisted therapy in the dental clinic may be an attractive alternative to sedation for anxious patients. Including a dental therapy dog in a clinical setting introduces new hazards and potential risks to health and safety for both humans and animal.

**Objectives:**

The study aims to describe potential hazards associated with risks to humans by having a therapy dog present in the dental clinic and to provide guidance on best practices to minimise and control risks for the patients, the dentist, and the dental clinic staff.

**Materials and Methods:**

Literature searches in Medline, http://Clinicaltrials.gov, and Google Scholar for qualitative and quantitative assessments of hazards and risks associated with the use of therapy dogs in health care settings, in combination with a review of the reference list of the included studies. Identified hazards and risks were analysed with respect for the health and welfare of humans in a dental clinic setting that involves the presence of a therapy dog.

**Results:**

Potential risks to health and safety for humans in dental clinics that offer dog‐assisted therapy can be categorised within four general categories of hazards: the dog as a source of zoonotic pathogens and human diseases, exposure to canine allergens, adverse animal behaviour, and dangers associated with high activity in a congested dental clinic operatory. Risks to humans are reduced by maintaining awareness amongst the dental clinic staff and the dog handler of all potential hazards in the dental clinic, and on how to reduce these hazards as well as adverse events that may scare the dental therapy dog.

**Conclusions:**

Risks to the health and safety of humans in the presence of therapy dog in the clinics are present but are low if the dental clinical staff and dog handlers comply with best practices.

## 1 INTRODUCTION

Dog‐assisted therapy (DAT) is one amongst several animal‐assisted therapy practices implemented in patient management scenarios in health care settings. Recent systematic reviews conclude that many patients benefit from interventions complemented with DAT (Kamioka et al., 2014; Lundqvist, Carlsson, Sjödahl, Theodorsson, & Levin, 2017; Waite, Hamilton, & O'Brien, 2018). These observations are encouraging, but an overriding concern is that therapy dogs in health care settings introduce new hazards and potential risks to health and safety. These hazards and potential risks must be identified, analysed, and minimised.

Hazards and potential risks must also be assessed for situations when any service dog, alternatively labelled as assistance or support dog, accompanies the patient in a health care facility. Service dogs are trained to work or perform tasks for the owner with a disability or to assist the impaired with difficulties effecting daily life tasks. Service dogs have therefore bestowed access rights to public offices and general hospitals in many countries. A service dog that accompanies its owner into a health care facility works in an unfamiliar environment, in contrast to therapy dogs that are specifically trained to work within the facility with the objective to provide comfort and psychological support to patients. Moreover, a therapy dog is always teamed together with a dog handler, whose sole task is to guide and observe the therapy dog to achieve optimum utility and risk mitigation. Published risk assessments relative to the presence of a service dog or a therapy dog in a general hospital facility focus principally on three potential hazards for humans. These are estimated potential risks of transmission of zoonotic pathogens, possible cross‐contamination, and exposure to canine allergens, typically in the context of patient populations with increased vulnerability (Linder, Siebens, Mueller, Gibbs, & Freeman, 2017; Murthy et al., 2015). An additional risk predominantly in nursing homes is injuries associated with falling or tripping over a dog (Stull, Hoffman, & Landers, 2018).

The risks of transferring antimicrobial resistant pathogens or antimicrobial resistance genes are a cause for constant vigilance. Multidrug‐resistant bacteria such as extended‐spectrum beta‐lactamase‐producing *Escherichia coli*, methicillin‐resistant *Staphylococcus aureus*, and methicillin‐resistant *Staphylococcus pseudintermedius* can be found in dogs and potentially transmitted to humans by direct contact or indirectly through public spaces (Damborg et al., 2016). Hitherto, the likelihood of transfer from domestic pets to humans appears to be minimal in health care facilities and in the general environment per risk assessments recently conducted in Germany (Feßler et al., 2018), Australia (Worthing et al., 2018), and Norway (VKM, 2015). However, contact with a therapy dog has been associated with increased risk of acquisition of methicillin‐resistant *S. aureus* colonised children. Although the incidence and impact of transmission of pathogens is unknown, some potential risk is apparent (Dalton et al., 2018; Lefebvre & Weese, 2009).

DAT has gained some interest in dentistry because patient anxiety is common and alternative interventions to reduce anxiety by sedation or provide care under general anaesthesia encompass risks (Cajares, Rutledge, & Haney, 2016; Havener et al., 2001; Manley, 2016; Schwartz & Patronek, 2002). The potential risks that therapy dogs pose to hospital staff and patients in general health care facilities (Lefebvre et al., 2008: Murthy et al., 2015; Linder et al., 2017) apply to various extents also in a dental clinical setting. However, additional hazards and potential risks apply in a dental clinical setting, and there is a need to identify all potential hazards associated with introducing a therapy dog into a dental clinical setting and to assess potential risks to health and safety.

The objective of this paper is to describe hazards associated with potential risks to health and safety to humans in dental clinics that have adopted DAT and to provide guidance on how to minimise and control risks for the patients, the dentist, and the dental clinic staff. A companion paper describes hazards associated with potential risks to health and safety to the dental therapy dog (Gussgard, Weese, Hensten, & Jokstad, 2019).

## 2 MATERIALS AND METHODS

The authors performed literature searches in http://Clinicaltrials.gov, Medline, and Google Scholar for use of therapy dogs in different health care settings. http://Clinicaltrials.gov listed 24 ongoing or completed studies on animal‐assisted therapy but only one on therapy dog use in dental care settings. The search strategy in Medline through http://Pubmed.com was modified from existing systematic reviews on animal‐assisted therapy using the following terms: (Dog OR canine) AND (Animal‐assist* OR Dog‐assist* OR Pet‐assist* OR Canine‐assist* OR animal‐therap* OR dog‐therap* OR pet‐therap* OR canine‐therap* OR “animal visitation” OR “dog visitation” OR “pet visitation” OR “canine visitation” OR therapy‐dog OR visiting‐dog). No study type, time limitation, sample size, or language filters were used. The search yield was *n* = 437 articles. Combining the search strategy with the search term (risk* OR hazard*) yielded *n* = 38 papers, alternatively with (dentistry OR dental) resulted in *n* = 7 papers. The reference list of the identified papers were further scrutinised to see if there were other relevant articles that should be appraised. Identified hazards and risks for humans associated with DAT in different health care settings were critically appraised with respect to their possible relevance to patient safety, as well as workplace health and safety in a dental clinic setting.

## 3 RESULTS

### 3.1 Hazards and potential risks in a dental clinical setting

Four general categories of hazards that involve potential risks to health for humans have been identified, that is, (a) the dog as a vector for zoonotic pathogens and human diseases, (b) exposure to canine allergens, (c) adverse animal behaviour, and (d) hazards associated with high activity in a congested dental clinic operatory.

#### 3.1.1 The dog as a vector for zoonotic and human pathogens

Like all other animal species, dogs harbour abundant and diverse bacteria, fungal, viral, and parasitic microbiotas. Included in these can be a range of potentially zoonotic pathogens. It is reasonable to assume that virtually every dog harbours one or more potentially zoonotic pathogens; however, the incidence of dog‐associated zoonotic infection is likely very low in light of the abundant dog–human contact that occurs. Regardless, although the incidence of zoonotic pathogen transmission from any individual dog–human encounter is very low, zoonotic diseases from dogs occur and the symptoms range from mild and self‐limiting to potentially peracute and fatal.

##### Hazards

A range of hazards have been identified in different health care facilities (Lefebvre et al., 2008: Murthy et al., 2015; Linder et al., 2017: Stull et al., 2018), of which some apply to a dental clinical setting.

Pathogens may be transferred from an infected dental therapy dog if the patient becomes exposed to any body fluids such as saliva or mucus, or by petting or touching the dog.

Accidental bites and scratches may be potentially high incidence problem.

A dental therapy dog risks transmitting an infectious pathogen from one human to another if the former has direct contact with the dental therapy dog, for example, by stroking the dog's fur.

##### Risk assessment

**Figure 1:**
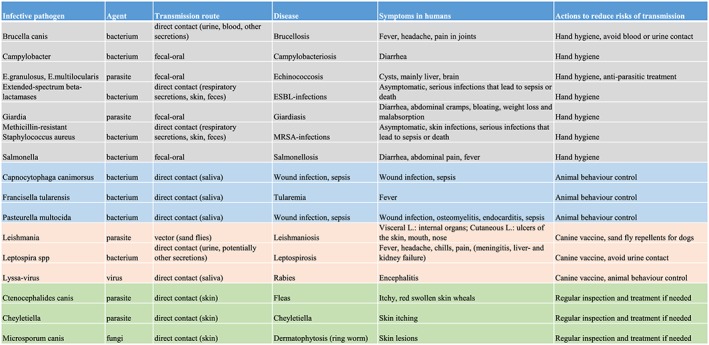
Precautionary actions to minimise risk of transmission of zoonotic pathogens from a dental therapy dog, examples of some of the most relevant zoonotic pathogens in a dental clinic setting

Some zoonotic pathogens can be transmitted from dogs, which include also from dental therapy dogs unless precautions have been done. A zoonotic infection may vary from mild symptoms to life‐threatening disease (Figure 1).

The probability of pathogen transmission from a dental therapy dog is indirectly influenced by the underlying prevalence of such pathogens amongst dogs in the local geographic area, which may vary considerably. Prevalence estimates depend on the host population constitution, the density of dogs that may be definitive or intermediate hosts, seasonal variation, regional, and local variances, and estimations can vary with the sampling methods and analytic techniques used to test for seroprevalence.

In some regions, the incidences of zoonotic diseases are very low, for example, in Norway (Norwegian Veterinary Institute, 2017), in contrast to other regions. Examples of regional variations in Europe of fecally transmitted diseases such as *Echinococcosis*, *Giardia*, and *Toxocara* ranges from 0.5% up to 30% (Baneth et al., 2016). These numbers include both stray dogs and more domesticated dogs. The bottom line is that there is a latent potential risk, but it is difficult to estimate explicitly how much.

Single dog hair shed by a well‐cared animal is not regarded as a hazard from an asepsis perspective, although dog hair has the capacity to harbour eggs of the parasite nematode *Toxocara canis*, and stray dogs represent a risk for transmission of the zoonosis. In dogs receiving correct care regarding hygiene, the risk of transmission of toxocariasis by direct contact or from hair shedding is considered as low (Merigueti et al., 2017).

##### Risk minimisation and proposed best practices

General guidance to minimise potential risk of transmission of zoonotic infections from companion animals to the public have been published (Day, 2016; Stull, Brophy, & Weese, 2015), and these apply also to practices in health care facilities including a dental clinical setting.

Most of the best practices to reduce potential risks of spread of zoonotic pathogens in health care facilities (Lefebvre et al., 2008; Murthy et al., 2015; Linder et al., 2017: Stull et al., 2018) are also feasible in a dental clinical setting. All universal guidelines regarding hygiene and protocols to avoid cross‐contamination in the dental clinic operatory must be strictly followed (Laheij, Kistler, Belibasakis, Välimaa, & de Soet, 2012; CDC. Centers for Disease Control and Prevention, 2019). Adverse events should be recorded and subjected to analyses for subsequent improvement. Emphasis relative to a dental therapy dog working in a dental clinical setting is that

- hand hygiene before and after sessions with the dental therapy dog is mandatory for the patient, the therapy dog handler, and the dental clinic staff;
- the patient should avoid direct contact with the dental therapy dog's saliva and mucous membranes;
- the patient should not be given the opportunity to offer treats to the dental therapy dog;
- the dental therapy dog must be regularly visually inspected for fleas and ticks and open wounds;
- the dental therapy dog requires health checks regularly by a veterinarian and must comply with required vaccines and deworming therapies;
- the dental therapy dog should not be fed with raw meat;
- the dental therapy dog should be discouraged from licking the patient;
- the dental therapy dog should be washed and bathed before and occasionally also after work in the dental clinic operatory;
- the dental therapy dog should wear socks to avoid unintentional scratches from the dog's claws, as well as to protect the chair upholstery; and
- the dog handler must control the dental therapy dog to avoid accidental bites or scratches.

Despite the minimal risk of transmission of zoonotic pathogens when practices comply with precautionary steps, it is important to recognise that some individuals are more susceptible to zoonotic diseases (Stull et al., 2014). Special considerations are required for considering the use of a dental therapy dog for children below the age of 5 years, frail elderly, women who are pregnant, individuals with any indwelling medical device, or immunocompromised persons. Although the dental clinician always should be aware of the general health condition of their patients, it is specifically incumbent to inquire patients considered to be exposed to a dental therapy dog about the latest medical status and drug intake.

#### 3.1.2 Exposure to allergens

##### Hazards

All dogs release proteins into their surroundings through secretions, as excretions, or as dander. One major allergen is a salivary lipocalin protein designated Can f1. Saliva contains three additional lipocalin allergens, that is, Can f2, Can f4, and Can f6, whereas serum contains an albumin named Can f3, and urine includes a prostatic kallikrein protein named Can f5. Until recently, Can f1 was considered as the principal dog allergen, but recent studies suggest that Can f5 may have been underestimated (Basagaña et al., 2017).

##### Risk assessment

The concentration of dog allergens is likely higher in a dental clinical setting involving DAT in comparison with a home without any pets. Be that as it may, every type of human indoor environment contains dog allergens, regardless of the presence of pets because dog allergens are transferred passively via clothing (Zahradnik & Raulf, 2014).

Allergen concentrations measured in health care facilities that are comparable with a dental clinical setting are lacking. One study reports indoor allergen concentrations in an animal hospital with great variations as a function of locations, leading the investigators to suggest that the air concentrations likely reflected the practice of vigorous and frequent cleaning in the operating room between the patient sessions (Samadi et al., 2010).

The potential consequences for humans that react to canine allergens are transient rhinoconjunctivitis, respiratory symptoms, rash, and potential acute urticaria, although the diagnosis of dog allergies is complex (Chan & Leung, 2018; Davila et al., 2018).

Critical allergy reactions, including anaphylaxis, related to any dog encounter has, to the authors' knowledge, never been reported in the scientific literature.

##### Risk minimisation and proposed best practices

- Currently, there is no reliable vaccine or subcutaneous immunotherapy available for dog allergy.
- Airborne dander from dental therapy dogs can be reduced by the use of high‐efficiency particulate air filters.

##### Dog breed

- A “hypoallergic dog” labelling is controversial, because different measurements are cited. Data exist on concentrations in reservoir dust and airborne allergen levels in the homes of dog owners, whereas other data focus on concentrations of allergens in samples from the fur (Vredegoor, Willemse, Chapman, Heederik, & Krop, 2012) or in the saliva (Breitenbuecher et al., 2016; Polovic et al., 2013).
- Hair shedding differs amongst different breeds, and the extent of accumulated hair shedding over time may be a more relevant variable for allergen contribution to the immediate environment than the actual concentrations of allergens in the hair. The length of hair does not seem to play a significant role regarding allergens (Heutelbeck, Schulz, Bergmann, & Hallier, 2008).

##### Practices

- The dental therapy dog should be newly bathed to remove loose dander prior to entering the dental clinical setting. Special shampoos are advertised as more effective than other for removing dander, but scientific data are lacking.
- The dental therapy dog should be prevented from licking the patient, and the patient should be encouraged to not incite facial licking (Saliva contains the Can f1, Can f2, Can f4, and Can f6 allergens).
- The dental therapy dog must be inspected regularly to ensure that the dog has no open wounds (Sera contain the Can f3 allergen).

#### 3.1.3 Animal behaviour

##### Hazards

Dog bites are common in the community and can be associated with disease risks and trauma and can occasionally be severe. Bites can occur for many reasons, including dominance aggression and fear. A dental clinic environment can be associated with atypical sights, noises, and activities, which could incite stress and potentially increase a tendency to bite in some dogs. Intensification of stress leads to disturbed behaviour, which may begin with whining and growling and escalate to clawing, escape manoeuvres, and biting at worst.

##### Risk assessment

A stressed dog that attempts to evade may tumble over objects or become entangled in chairs or instruments and risks injuring the humans in the dental clinic operatory as well as inflicting self‐injury.

A dog bite is an emotional trauma besides carrying a latent risk of zoonotic pathogen transmission or wound infection.

##### Risk minimisation and proposed best practices

- Any dog considered for use as a dental therapy dog must undergo a proper temperament testing, done in a structured manner by someone with adequate expertise.
- The dental therapy dog should be examined and evaluated regularly by a veterinarian, to avoid disturbed behaviour due to pain or sickness. Any physical discomfort may lead to a change in the dog's behaviour or capability to work as a dental therapy dog.
- The dental therapy dog team, that is, the dental therapy dog and its handler, must undergo regular re‐evaluation, training, and recertification as a team.
- The dental therapy dog should have access to a separate room for resting and recuperating undisturbed between patients. This will minimise the chance of the dental therapy dog becoming stressed due to a work overload.

Forestalling stress and disturbed behaviour are essential. At least five factors must be considered, which include (1) the competency of the dog handler and the demeanour of the dental therapy dog, (2) the intended function of the dental therapy dog during the patient treatment, (3) the patient preference regarding the location of the dental therapy dog, (4) the delegated responsibilities for monitoring the dental therapy dog during the treatment session, and (5) monitoring all adverse events.

1. Adequate training of the dental therapy dog and its handler and dog demeanour

- Any dog considered as a therapy dog should pass a temperament test before embarking on a training course. A trained therapy dog is a confident dog and therefore performs as anticipated with a low likelihood of an adverse behaviour. A trained dog handler can immediately identify first signs of adverse behaviour. Best performance is when a therapy dog and its handler have been trained jointly as a team. Training centres offer thorough training programmes and examinations for certification with requirements for recertification. Additional training is required for a dental therapy dog because the need for specially training in a dental clinic operatory, which includes familiarity with all distinct characteristics such as smell, sound, and movements of the dental patient chair.
- The dental therapy dog must not be frightened by sudden noise from the patient or generated by common dental procedures such as the use of the vacuum suction, or a dental drill, or ultrasound scaling for removing calculus. A typical experience with anxious patients is a loud wail when experiencing pain. The dog handler should be familiar with the different clinical procedural steps, so he or she can be prepared to distract the dental therapy dog in case of sudden noise that may distress the dental therapy dog. An optimal arrangement may be a dog handler with a dental training background, for example, a licensed dental therapist, dental hygienist, or dental assistant. Further benefits of such solution are a likely better compliance with cross‐infection control measures and adherence to legislative patient confidentiality regulations.

2 Intended function of the dental therapy dog in the clinic

**Figure 2:**
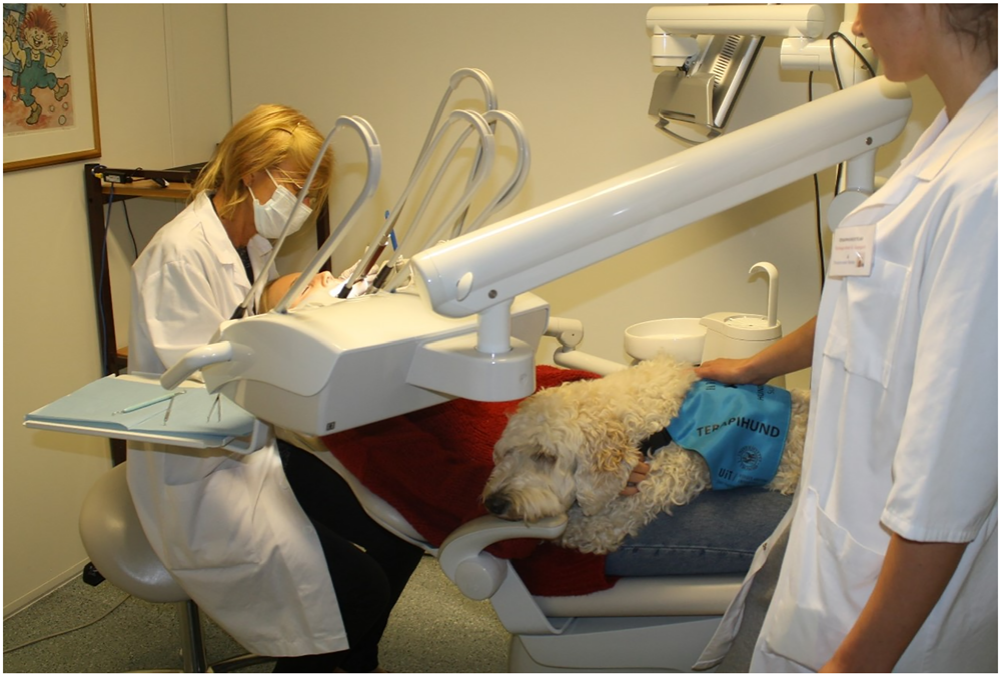
The dentist concentrates fully on providing best patient care while a dog handler constantly monitors the dental therapy dog for signs of discomfort. Communication between the three stakeholders is essential. Photo: A. M. Gussgard

- The dentist must, for every patient situation, determine who should be responsible for monitoring the dental therapy dog once the patient is seated. Alternatives are the dentist, a dental clinic staff, or a separate dog handler. For most treatment procedures, it is not possible or appropriate for the dentist to focus on both the dental therapy dog and the patient simultaneously, hence necessitating a separate individual to monitor the dental therapy dog (Figure 2). Selecting who to delegate this responsibility must take into consideration not only the patient characteristics but also the type of dental intervention including exposure to potentially hazardous materials and substances.

3 Patient preference regarding the location of the dental therapy dog

**Figure 3:**
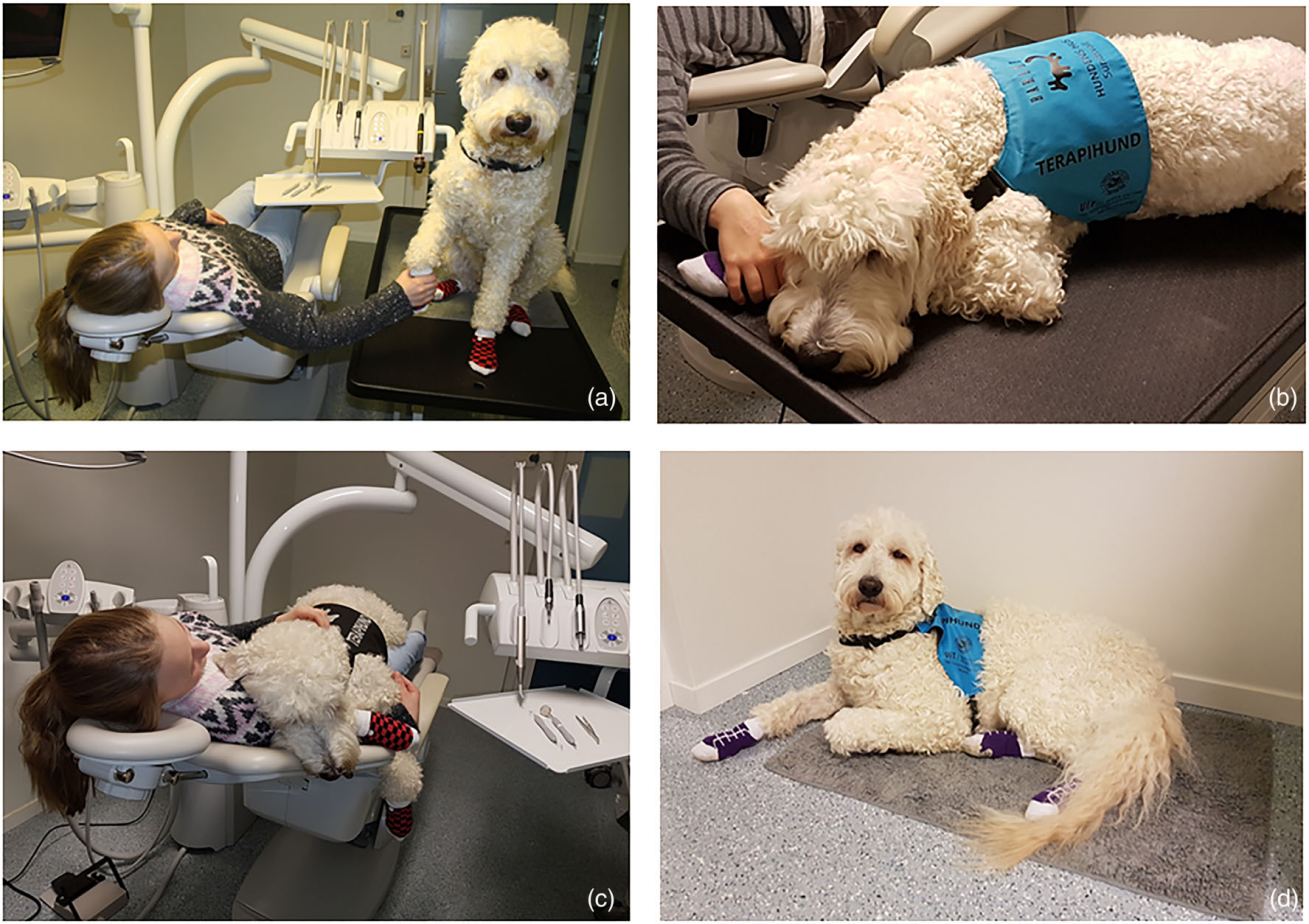
Alternative locations for the dental therapy dog. Positioned on a veterinary table adjacent to the patient chair (a,b), positioned on patient lap in the patient chair (c), positioned on a carpet in the corner of the dental clinic operatory (d). Photo: A. M. Gussgard

- For some dental procedures, it is advisable that a distance is maintained between the dental therapy dog and the clinician–patient, but for most procedures, the location of the dental therapy dog is optional and left to the patient to decide.
- Some patients prefer having the dental therapy dog positioned on their lap while being seated in the dental chair. Other patients prefer having the dental therapy dog positioned on a veterinary table adjacent to the patient chair for easy reach. A third option is when the patient is satisfied if the dental therapy dog remains in the dental clinic operatory, for example, on the floor (Figure 3).

4 Delegated responsibility for monitoring the dental therapy dog

- The location of the dental therapy dog, the number of personnel involved in the dental treatment, and the dental procedure performed allow for alternative scenarios regarding responsibility. It is important that once the responsibilities have been delegated that there are no misunderstandings.
- The dog handler must constantly monitor the dental therapy dog to avoid any adverse events.
- The dog handler must act at first signs of stress or exhaustion in order to avoid a stress‐induced unwanted behaviour.

5 Adverse event monitoring

- An adequate reporting mechanism for adverse events is desirable. Adverse events need to be monitored and critically appraised with the objective to prevent or mitigate subsequent undesirable situations. For example, any manifestation of a fearful or a dominant response to a situation, even if it did not lead to any harm, needs to be investigated to ensure that the presence of the dental therapy dog and its handler in the dental clinic operatory are acceptable from a risk perspective.

#### 3.1.4 High activity in a congested dental clinic operatory

##### Hazards

A dental clinic operatory may be quite small with the dentist seated intimately next to the patient who remains confined horizontally in an examination chair surrounded by an instrument table and flexible hoses located above the patient's chest. The floor space adjacent to the dental chair is often limited to enable the dentist and the clinic staffs to readily find instruments and materials that are required, while maintaining strict adherence to an infection‐control regimen.

A treatment session can last anywhere from 10 min to several hours, depending on the type of patient management and intervention, with or without the presence of the clinic staff throughout the treatment session. Immediately after the session completion, the clinic staff removes all contaminated tools and waste according to protocol, and the dental unit and adjoining areas are cleaned, disinfected, and prepared for the next patient. All unplanned delays are disruptive and stressful for all stakeholders.

##### Risk assessment

A dental therapy dog in a congested dental clinic operatory where the activity is high can cause someone accidentally tripping and experiencing a fall injury.

The size of the dental therapy dog and its position influences the risk of failing to see the dog. Small size dogs and dogs positioned on random floor locations rather than on a dedicated veterinary table or a specific spot in a corner of the dental clinic operatory increase the risks.

##### Risk minimisation and proposed best practices

**Figure 4:**
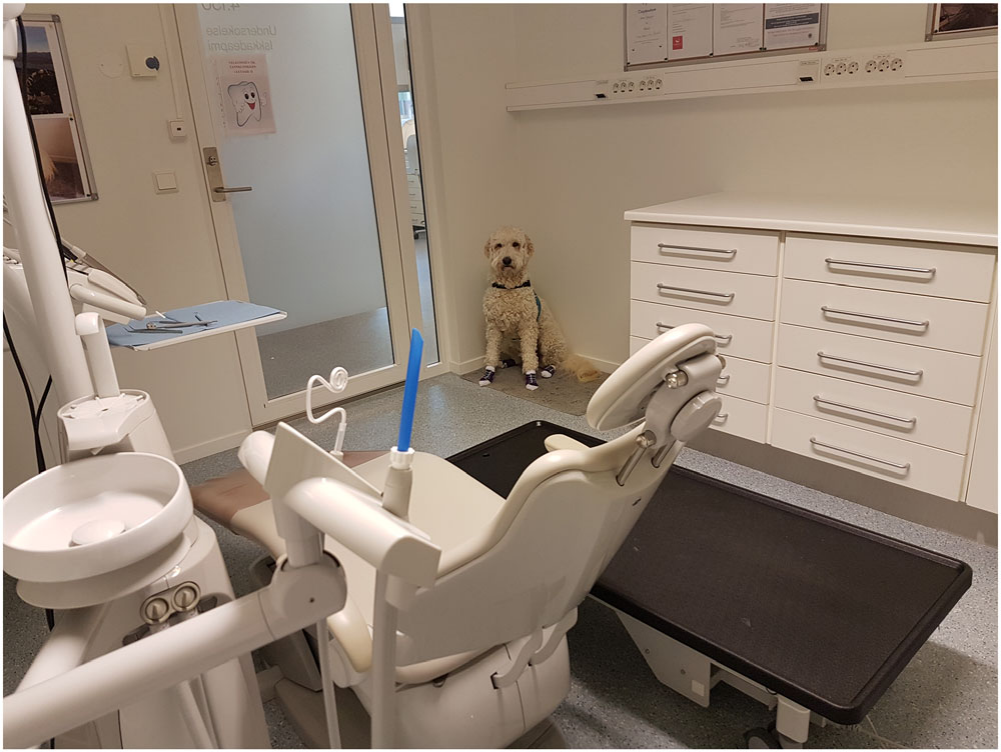
The dental therapy dog should be trained to go to a safe spot (e.g., a dog‐carpet in a corner of the dental clinic operatory) and to stay put until further notice. Photo: A. M. Gussgard

- When the dental therapy dog enters or exits the dental clinic operatory, the dog handler must focus on where the dental therapy dog is placed and, if necessary, protect the animal from being stepped on or tumbled over by the patient or the dental clinic staff. The dental clinic staff must also take responsibility for protecting the patient from falling over the dog.
- Teaching the dental therapy dog to move on command to a safe spot in the room (e.g., a dog‐carpet on the floor in the room corner) and to stay put until further notice is useful to make sure that the dog can leave and stay out of the traffic zone (Figure 4).

## 4 DISCUSSION

In a dental clinical setting, the legal responsibility for providing best patient care and not expose patients to unnecessary risks rests solely with the dentist. Some precautions are required if the dentist considers bringing in a dental therapy dog into his or her dental clinical setting.

Not all dogs are suited to work as a dental therapy dog, and not all humans are suited as a dog handler. A dentist must conduct a critical evaluation of the anticipated dental therapy dog as well as its handler and request documentation of completed training course for the dental therapy dog teams. Critical elements are the assessment of the dog's temperament before and during the training course, the contents of the curriculum of the training course, the quality of its teachers, and their evaluation of the dental therapy dog team. Modern training of dogs should focus on positive reinforcement, whereas punishment should not be part of teaching on how a dog should behave. A general certification as a therapy dog, without any specific working objectives, may certify that this dog (and handler) has obtained certain basic knowledge and training, but this should not be enough to work as a dental therapy dog in a dental clinical setting. To reach the next step as dental therapy dog, further theory is necessary, and the therapy dog and its handler have to be trained in an environment where the animal is supposed to work. By assuring that the dental therapy dog and the dog handler have undergone a proper education and that the dog handler brings the dog for regularly veterinary assessments, most likely there will not be any problems regarding control of the animal behaviour in the dental clinic. The dentist should also ask the dog handler to provide proof of regularly veterinary care, showing that all necessary vaccines or other medical treatments have been accomplished.

Universal guidelines regarding hygiene in the dental clinic, as well as proper hand hygiene, are mandatory for all individuals engaged in patient care. Although the hazards and potential risks described in the previous sections focus on the dental therapy dog, it is prudent to recognise that the dog handler may also pose a risk, especially if he or she has no training in health care provision, understanding of need for asepsis and adherence to hygiene regimes. The dog handler must also be considered as a possible source for pathogens, including multidrug‐resistant bacteria. Ultimately, it is the dentist that needs to safeguard that all guidelines and recommendations are being followed.

Where the dental therapy dog meets the patient needs some consideration. One alternative is that this occurs in the waiting area. One may consider posting a sign in the waiting room that explains that a dental therapy dog may appear occasionally. However, not all patients understand or even concur with the idea that a dental therapy dog is present for anxious patients. Moreover, some individuals may find it awkward to be singled out as above‐anxious in a room filled with other people.

Most human beings are capable of making their appointment with the dentist, and they may cope well during their dental appointment, even though it may not be considered as the most pleasant thing to do. For people who suffer from odontophobia, it may not be that easy. The dental therapy dog could certainly reduce stress and anxiety and also act as a communication icebreaker. It may be easier for an anxious patient to talk about his/her dental anxiety while petting the dog.

If the intention of the dental therapy dog is solely for communication purposes, maybe the consultation can take place in an office next to the dental clinic operatory, that is, not in a room where there is dental equipment, chemicals, and so on. If an ordinary room is used, the extra risks and safety issues regarding high activity in a congested dental clinic operatory are minimised.

Mainly for children, but perhaps also for adults, the dental therapy dog can help in teaching a patient to sit in the dental chair by merely training the dental therapy dog to jump into the dental chair on command and stay calm when the chair is taken into the correct position for a dental examination. The dental therapy dog should also be accustomed to a dental examination and thereby be able to demonstrate for the patient that there is nothing to worry about. When the dental therapy dog shows comfort and calmness in the dental chair, the child may want to try itself. If the dental therapy dog is being solely used as a demonstrator, there is less need for dental chemicals, sharp instruments, and so on that may harm the dog, and there is less need for close contact with the dog and the patient; hence, the risks both for humans and for the dental therapy dog are reduced.

## 5 CONCLUSION

All hazards in the dental clinic are challenges for the dentist, the dental clinic staff, and the dental therapy dog team. However, with a proper risk assessment and appropriate routines, the chance of any adverse events seems low. The hazards in the dental clinics that have been described in this paper are real. However, all the risks that we have identified and appraised are conceivable risks, and currently, there is no scientific data to substantiate whether the rates of the individual risks may be considered as low, medium, or high. The potential benefits of adding DAT into a dental clinical setting needs to outweigh the risks for all involved humans and for the dental therapy dog.

## CONFLICT OF INTEREST

None declared.
